# Fine scale geographic residence and annual primary production drive body condition of wild immature green turtles (*Chelonia mydas*) in Martinique Island (Lesser Antilles)

**DOI:** 10.1242/bio.048058

**Published:** 2019-12-09

**Authors:** Marc Bonola, Marc Girondot, Jean-Patrice Robin, Jordan Martin, Flora Siegwalt, Lorène Jeantet, Pierre Lelong, Clément Grand, Philippine Chambault, Denis Etienne, Julie Gresser, Gaëlle Hielard, Alexandre Arqué, Sidney Régis, Nicolas Lecerf, Cédric Frouin, Fabien Lefebvre, Emmanuel Sutter, Fabien Vedie, Cyrille Barnerias, Laurent Thieulle, Robinson Bordes, Christelle Guimera, Nathalie Aubert, Myriam Bouaziz, Adrien Pinson, Frédéric Flora, Matthieu Duru, Abdelwahab Benhalilou, Céline Murgale, Thomas Maillet, Lucas Andreani, Guilhem Campistron, Maxym Sikora, Fabian Rateau, Francis George, Joffrey Eggenspieler, Thierry Woignier, Jean-Pierre Allenou, Laurent Louis-Jean, Bénédicte Chanteur, Christelle Béranger, Jessica Crillon, Aude Brador, Caroline Habold, Yvon Le Maho, Damien Chevallier

**Affiliations:** 1 Université de Strasbourg, CNRS, IPHC UMR 7178, F-67000 Strasbourg, France; 2 Laboratoire Écologie, Systématique, Évolution, Université Paris-Sud, AgroParisTech, CNRS, Université Paris Saclay, 91405 Orsay, France; 3 UMR MARBEC, IFREMER, CNRS, IRD, University of Montpellier, Avenue Jean Monnet, 34200 Sète, France; 4 DEAL Martinique, Pointe de Jaham, BP 7212, 97274 Schoelcher Cedex, France; 5 Office de l'Eau Martinique, 7 Avenue Condorcet, BP 32, 97201 Fort-de-France, Martinique, France; 6 Surfrider Foundation Europe, 97000 Fort-de-France, Martinique, France; 7 Association POEMM, 73 lot papayers, Anse a l'âne, 97229 Les Trois Ilets, Martinique, France; 8 ONF International, 78 route de Moutte, 97207 Fort-de-France, France; 9 Aix Marseille University, University Avignon, CNRS, IRD, IMBE, Marseille, 13397, France; 10 IFREMER Délégation de Martinique, 79 Route de Pointe-Fort 97231 Le Robert, France; 11 PNR Martinique, Avenue des Caneficiers, 97200 Fort-de-France, France; 12 Parc Marin de Martinique, Agence Française pour la Biodiversité, Avenue des Caneficiers, 97200 Fort-de-France, France

**Keywords:** Green turtles, Juveniles, Body mass, Body condition, Biometry

## Abstract

The change of animal biometrics (body mass and body size) can reveal important information about their living environment as well as determine the survival potential and reproductive success of individuals and thus the persistence of populations. However, weighing individuals like marine turtles in the field presents important logistical difficulties. In this context, estimating body mass (BM) based on body size is a crucial issue. Furthermore, the determinants of the variability of the parameters for this relationship can provide information about the quality of the environment and the manner in which individuals exploit the available resources. This is of particular importance in young individuals where growth quality might be a determinant of adult fitness. Our study aimed to validate the use of different body measurements to estimate BM, which can be difficult to obtain in the field, and explore the determinants of the relationship between BM and size in juvenile green turtles. Juvenile green turtles were caught, measured, and weighed over 6 years (2011–2012; 2015–2018) at six bays to the west of Martinique Island (Lesser Antilles). Using different datasets from this global database, we were able to show that the BM of individuals can be predicted from body measurements with an error of less than 2%. We built several datasets including different morphological and time-location information to test the accuracy of the mass prediction. We show a yearly and north–south pattern for the relationship between BM and body measurements. The year effect for the relationship of BM and size is strongly correlated with net primary production but not with sea surface temperature or cyclonic events. We also found that if the bay locations and year effects were removed from the analysis, the mass prediction degraded slightly but was still less than 3% on average. Further investigations of the feeding habitats in Martinique turtles are still needed to better understand these effects and to link them with geographic and oceanographic conditions.

## Abstract (precis)

**Summary:** This study validates the use of different body measurements to estimate body mass. We show a yearly and north-south pattern between them, strongly correlated with net primary production.

## INTRODUCTION

Animal physiological state is potentially related to evolutionary fitness. Health can be an indicator of past foraging success, fighting ability and the ability to cope with environmental pressures, any of which may ultimately impact reproductive success (Jakob et al., 1996). In the animal kingdom, the search for condition indices related to individual health and fitness has been a longstanding quest (Fulton, 1904; Le Cren, 1951; Stevenson and Woods, 2006). Indeed, body size is a structural characteristic that has a remarkable influence on fitness during life (Churchill et al., 2014; Damuth and MacFadden, 1990; Peters, 1983; Schmidt-Nielsen, 1984), especially on energy expenditure, reproduction behaviour, locomotion and community structuration in relation to habitat (Cardillo et al., 2005; Fariña et al., 1998; Lindenfors et al., 2002; Nee et al., 1991; Schmidt-Nielsen, 1984; Tuomi, 1980; Van Valkenburgh, 1990). The evolution of body size can thus reveal important information about the *in situ* environment specific to each species and be decisive in terms of the survival potential and reproduction success of a population (Clutton-Brock, 1991; Gaillard et al., 2000).

The comparison of the growth rates of different species of marine turtles living at the same site reveals that immature green turtles (*Chelonia mydas*) grow slower than hawksbills (*Eretmochelys imbricata*) and loggerheads (*Caretta caretta*) of a similar size (Bjorndal and Bolten, 1988). Food consumption at the scale of a population or an individual, energy fluxes through trophic levels, and ultimately a better understanding ecosystem functioning can be assessed using body mass (BM) growth analyses (Bjorndal and Bolten, 1988; Chaloupka and Musick, 1997; Price et al., 2004; Trites et al., 1997).

The relationship between body size and BM has been established in many studies on different species raised in laboratory conditions, zoos, or living in semi-free-range or natural environments (Smith and Jungers, 1997). Thus, precise estimations of BM in relation to body size are, for example, available in insects (Rogers et al., 1977; Schoener, 1980), spiders (Brady and Noske, 2006; Sage, 1982), birds (Boos et al., 2000; Viblanc et al., 2012), marine mammals (Trites and Pauly, 1998), and fishes (Froese and Palmares, 2000; Kohler et al., 1995; Martin-Smith, 1996).

Despite the fact that a precise estimation of BM can be used to determine growth rate in marine turtles, only a few studies investigating the relationship between body size and BM have been conducted to date. Studies of this relationship have been restricted to subadult and adult individuals in green turtle (Bjorndal and Bolten, 1988; Hays et al., 2002), hawksbill turtle (Santos et al., 2010) and leatherback turtle (*Dermochelys coriacea*) (Georges and Fossette, 2006). Determining the relationship between BM and body measurements, and more generally, studying their ecology and demographic evolution, is difficult for juvenile turtles due to their permanent life at sea at this stage (Bass and Witzell, 2000; Pelletier et al., 2003). Indeed, although capture-mark-recapture (CMR) is facilitated in adult females during the laying season (Casale et al., 2007), it is more complicated in immature individuals, because it requires capturing the animals directly at sea (Limpus and Chaloupka, 1997). Nevertheless, the Lesser Antilles Islands’ concentration of immature marine turtles presents a unique opportunity to study individuals at this early stage in life (Chambault et al., 2018). Indeed, in the seagrass meadow that develops on the coastal fringe of these islands, a significant number of individuals with particularly high site fidelity feed all year round. This fidelity to ecosystems rich in high-energy food resources facilitates CMR as well as the continuous observation of immature individuals. A previous study of immature green turtles showed that BM can be predicted with high accuracy based on carapace length (Bjorndal and Bolten, 1988). Our study aimed to validate the use of different body measurements to estimate BM, which can be difficult to obtain in the field, and explore the determinants of the relationship between BM and size in juvenile green turtles. We established several predictive equations to estimate the BM of immature green turtles according to different morphological measurements and study the ecological determinants of this relationship. The determinants of the relationship between BM and size are then explored using several oceanographic and geographic proxies.

## RESULTS

### BM and body size of individuals

Overall, 323 different green turtles were captured for a total of 412 captures and recaptures (Table 1). A total of 258 individuals were captured only once, 48 twice, 12 three times, 3 four times, and 2 five times.

**Table 1.**
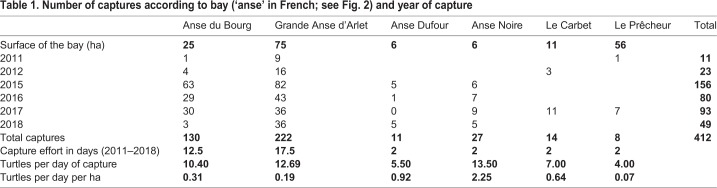
**Number of captures according to bay (‘anse’ in French; see Fig. 2) and year of capture**

Standard body measurements ranged from 26.0 cm to 93.8 cm for curved carapace length (CCL) and from 22.5 cm to 84.3 cm for central curved carapace width (CCCW). The lightest turtle weighed 2.2 kg and the heaviest 98.8 kg. Circumference at mid-carapace length (CmidCCL) ranged from 46 cm to 159 cm.

### Model for BM estimation with dataset A

**Fig. 1.**
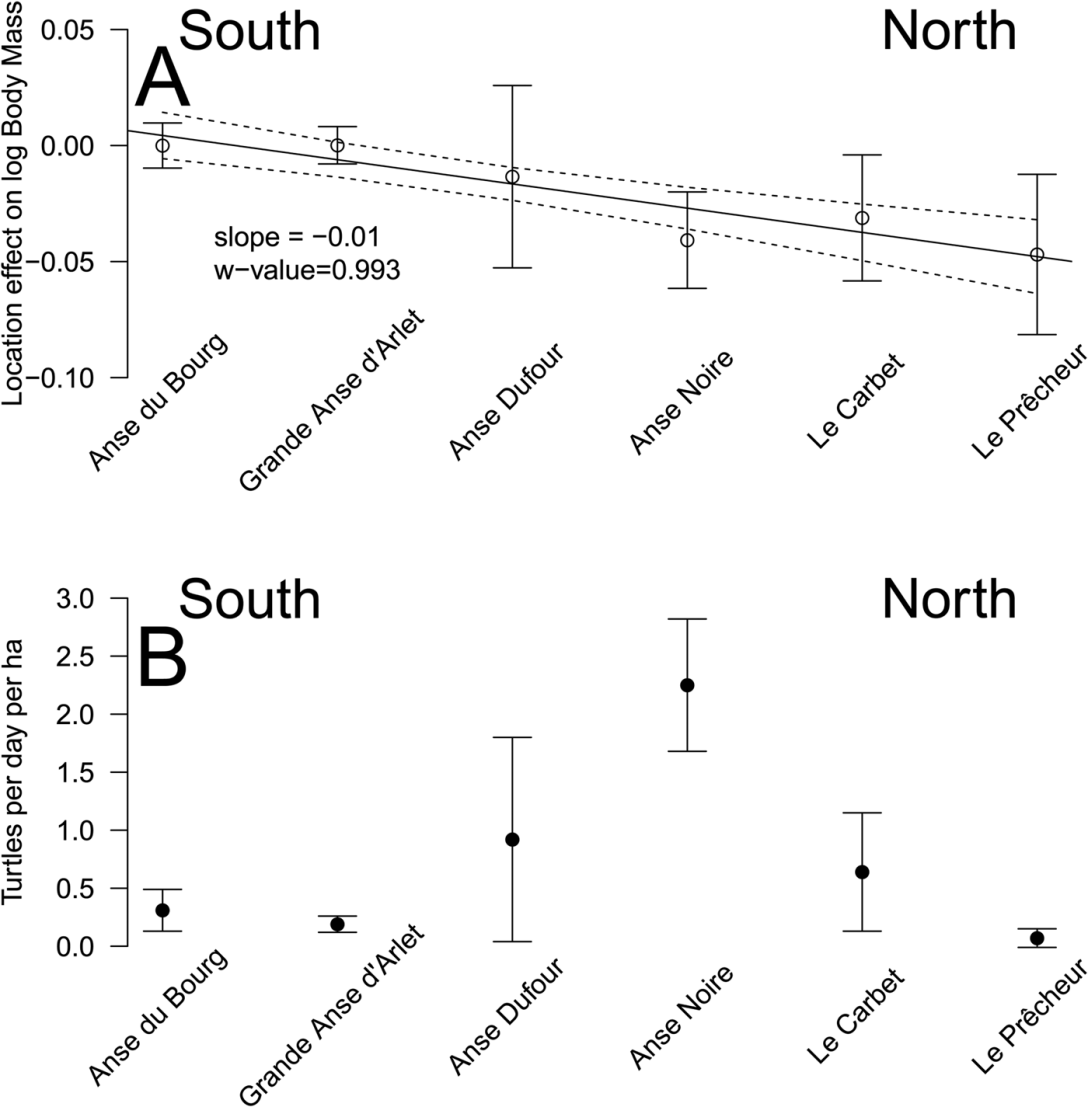
**(A) Bay location effect on log BM.** A negative value indicates that BM is lower than expected based on the size of the individual. Anse du Bourg was used as a reference and was thus equal to 0. Bay locations are ordered from south to north (left to right). Bars are the quasi-standard errors (Firth and de Mezezes, 2004). The significant south-north trend (1 for southernmost, 6 for northernmost location, slope=–0.01, w-value=0.993 being the posterior probability that the slope is different from 0) based on the linear model is shown along with its 95% confidence interval. If the distances between sites is used as regressors the conclusion is unchanged (slope=–0.002, se=0.001, w-value=5.513). (B) Density of turtles corrected for pressure of capture. Bars represent standard errors.

A total of 181 captures are considered in dataset A as the circumference was only measured in 2016, 2017 and 2018. The selected model to explain BM included CCL, CCCW, CmidCCL, the interactions between CCL, CCCW and CmidCCL, as well as the identity of the animal, year and location (Table 2). Its probability in being the best model among those tested was 0.45 according to the Akaike weight (Table 2). For example, a turtle being measured weighed 50 kg and based on its measurements it would be predicted to weigh between 49.15 and 50.85 kg with maximum and minimum being 45.3 and 54.6 kg, respectively. With this model, the average error for BM prediction was 1.70% (range=0%–9.22%) (Table 3). Using this dataset, a significant effect for the location of the bay where the turtles were caught was observed. When the six bay locations were ordered from south to north, a clear pattern emerged: turtles were lighter relative to their size in the northern bays (w-value=0.94; not shown for dataset A; see results for dataset B and Fig. 1 for a similar effect). The w-value is the posterior probability that a model with a slope different from 0 is better than a model with a slope fixed to 0 based on the Bayesian information criterion (Girondot and Guillon, 2018).

**Table 2.**
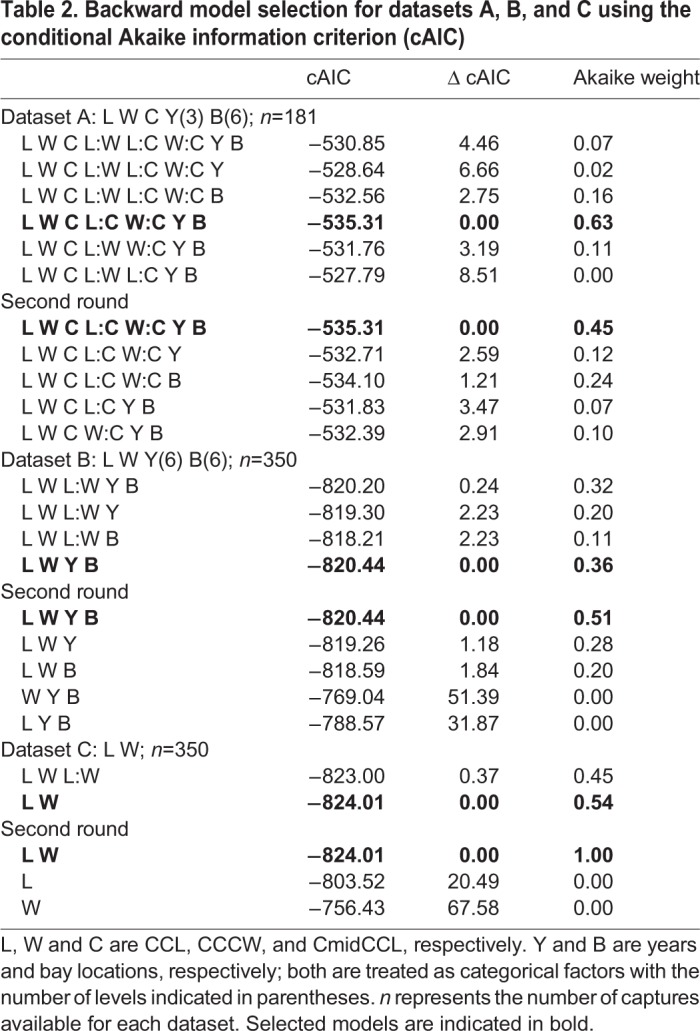
**Backward model selection for datasets A, B, and C using the conditional Akaike information criterion (cAIC)**

**Table 3.**
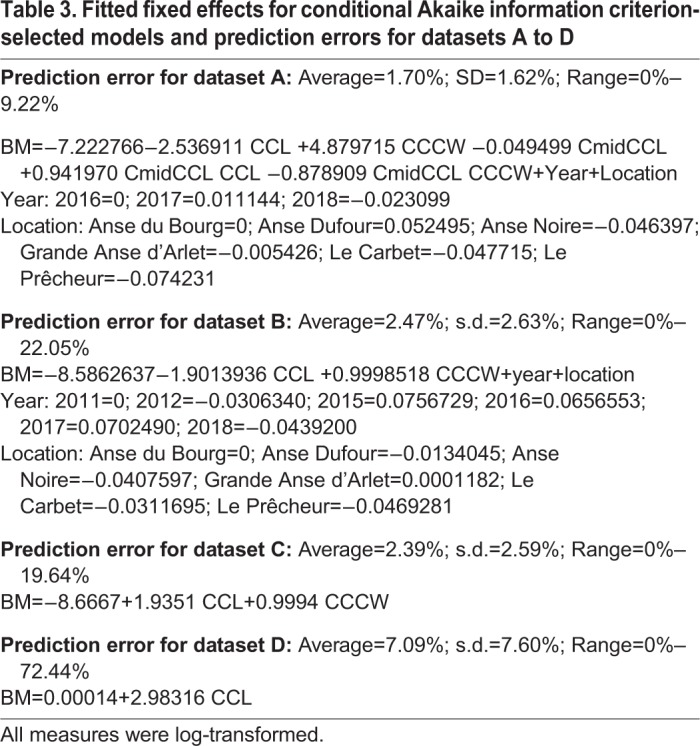
**Fitted fixed effects for conditional Akaike information criterion-selected models and prediction errors for datasets A to D**

### Model for BM estimation with dataset B

A total of 350 captures from 2011–2018 (6 years and six locations) constituted dataset B. The selected model included CCL, CCCW, as well as the identity of the animal, year and location. Its probability in being the best model among those tested was 0.51 according to the Akaike weight (Table 2). The second model without location effect had a support of 0.28. With the selected model, the average error for BM prediction was 2.47% (range 0–22.05%) (Table 3). For example, a turtle being measured weighed 50 kg and based on its measurements it would be predicted to weigh between 48.7 kg and 51.2 kg with maximum and minimum being 38.97 and 61.0 kg, respectively. Using this dataset, we observed the same significant effect of that of the location of the bay where the turtles were caught as seen in dataset A. When the bay locations were ordered from south to north, a clear pattern emerged; turtles were lighter relative to their size in the northern bays (linear model weighted by the inverse of quasi-standard error at each location, w-value=0.997; Fig. 1). An effect of year was also noticed, and turtles caught in 2011 and 2012 were significantly lighter relative to their size than those caught after 2014 (Fig. 3). This effect can also be seen in the pattern linking BM, CCL and CCCW according to the year of capture (Fig. 4).

**Fig. 2.**
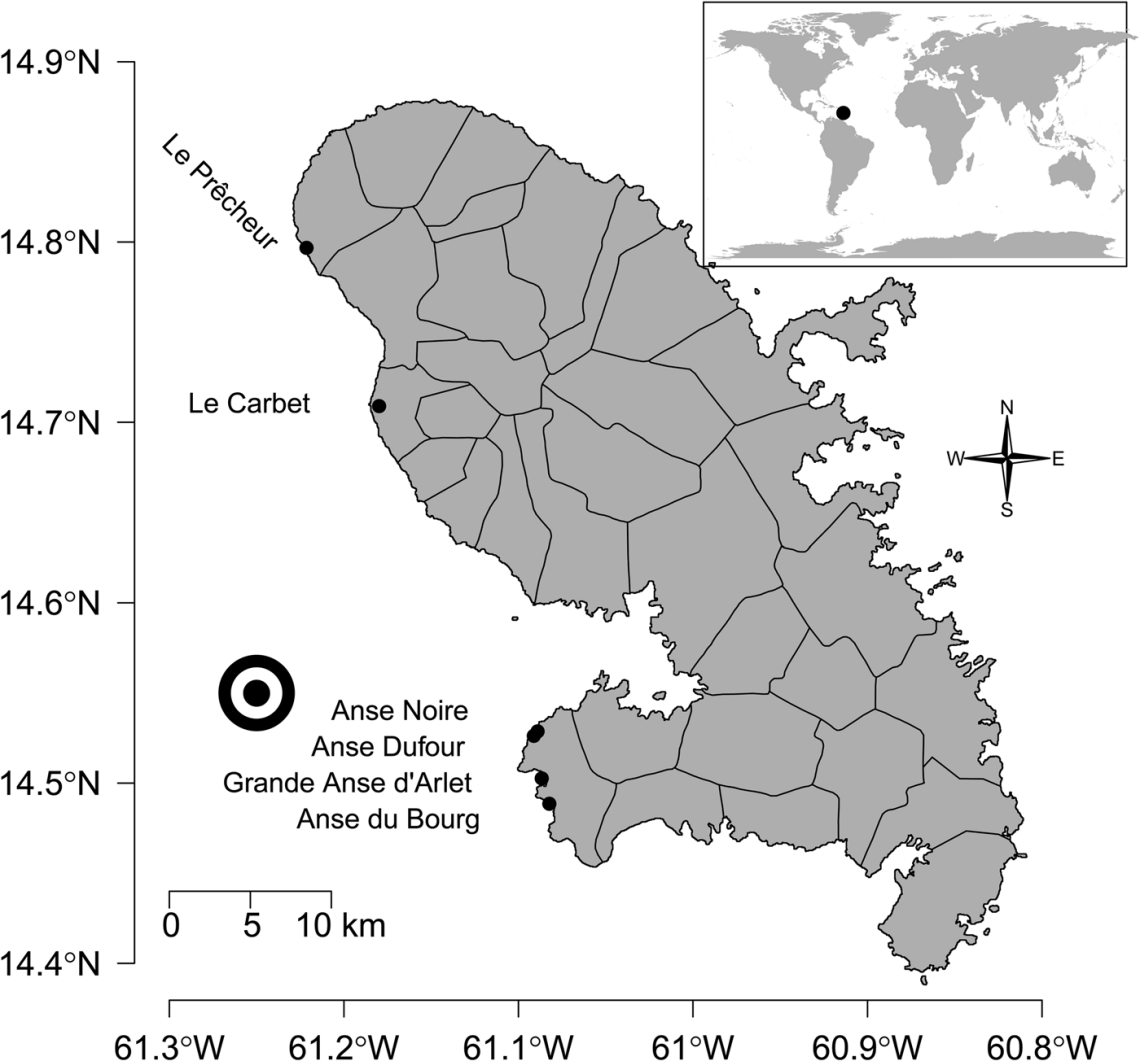
**Localisation of Martinique Island in the Caribbean Sea (top panel) as well as the bays where the turtles were caught (dots).** The black and white circles indicate the position where the net primary production, wind speed and sea surface temperature were measured (see Fig. 5).

**Fig. 3.**
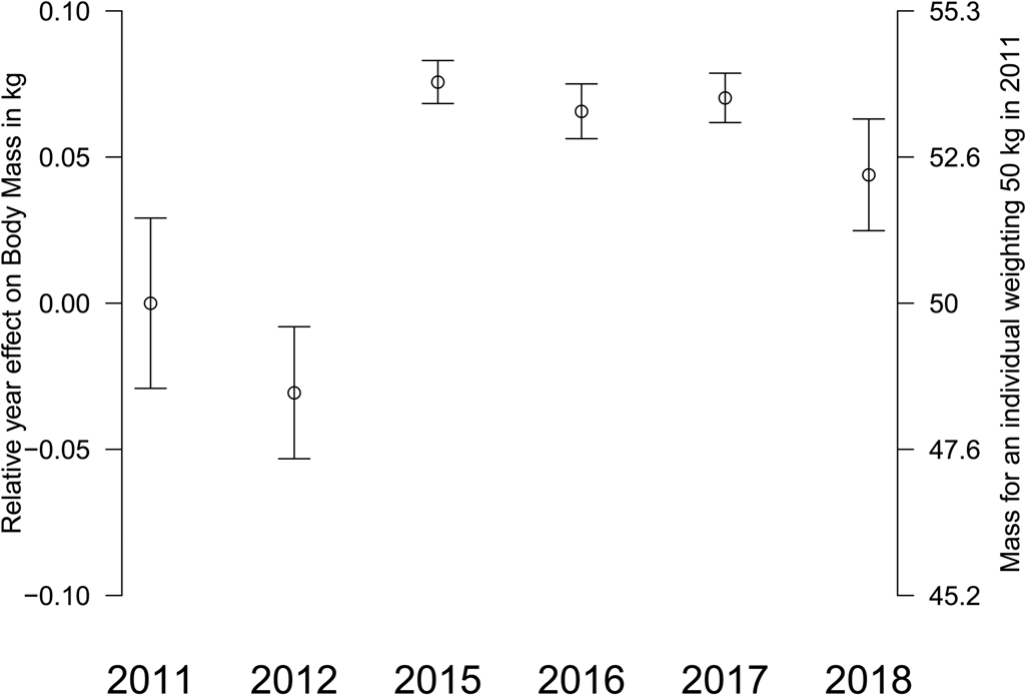
**Year effect on log BM for turtles captured near Martinique Island.** Bars are the quasi-standard errors (Firth and de Mezezes, 2004). A negative value indicates that BM was lower than expected based on the size of the individual. The year 2011 was used as a reference and was thus equal to 0.

**Fig. 4.**
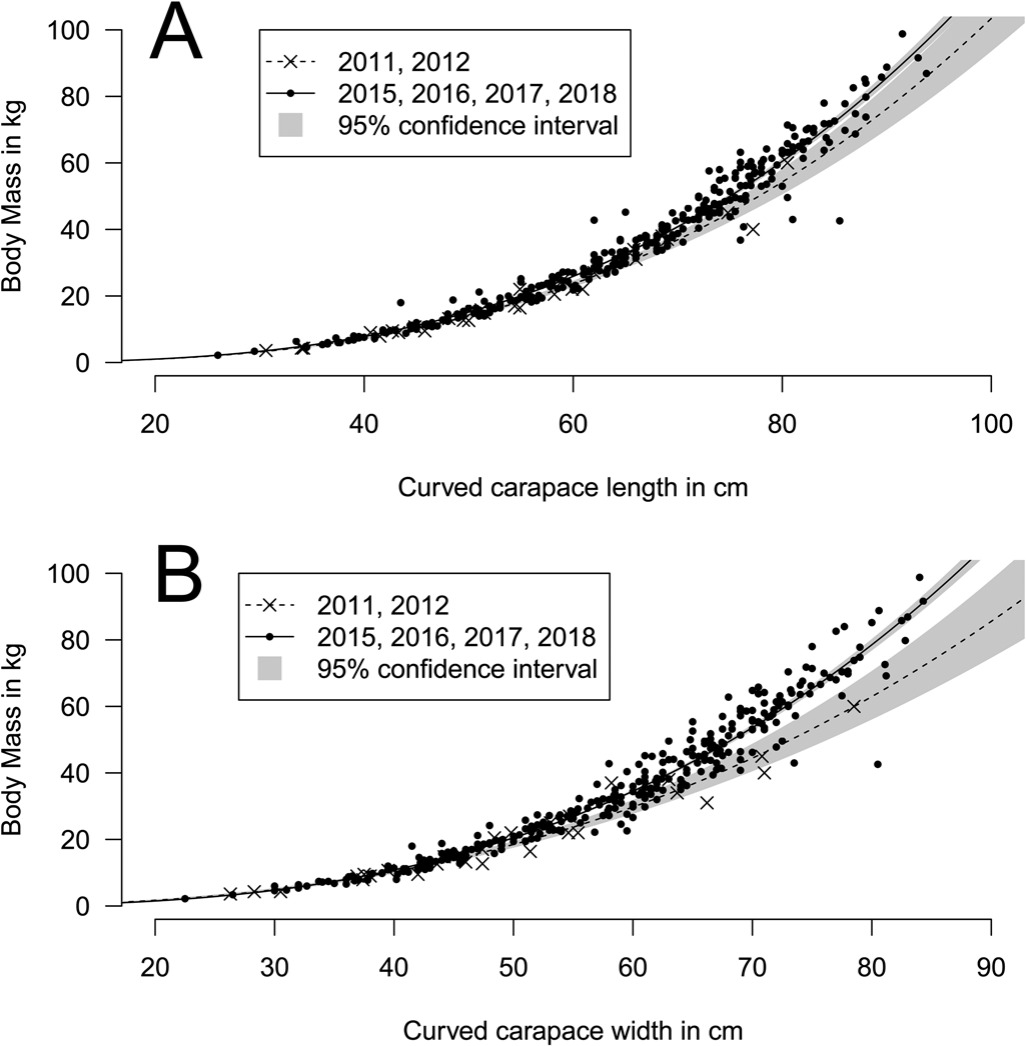
**Relationship between BM and (A) CCL and (B) CCCW for turtles caught in 2011 and 2012 (crosses) or from 2015 (points).** Crosses are located mostly at the bottom of the distributions, indicating that turtles are lighter than expected relative to their size in 2011 and 2012 (see also Fig. 3). Fitted model of log BM against log CCL and log CCCW as well as the 95% confidence interval (shaded area) are shown for both time periods.

### Model for BM estimation with dataset C

In this dataset, we considered the same turtles as in dataset B, although the selected model only included CCL, CCCW and the identity of animal. Its probability in being the best model among those tested was close to 1 according to the Akaike weight (Table 2). With this model the average error for BM prediction was 2.39% (range 0–19.64%) (Table 3). For example, a turtle being measured weighed 50 kg and based on its measurements it would be predicted to weigh between 48.8 kg and 51.1 kg with maximum and minimum being 40.18 and 59.82 kg, respectively. Aside from the detection of significant year and location effects (see results for dataset A and B), the prediction of BM with or without these effects was similar.

### Model for BM estimation with dataset D

**Fig. 5.**
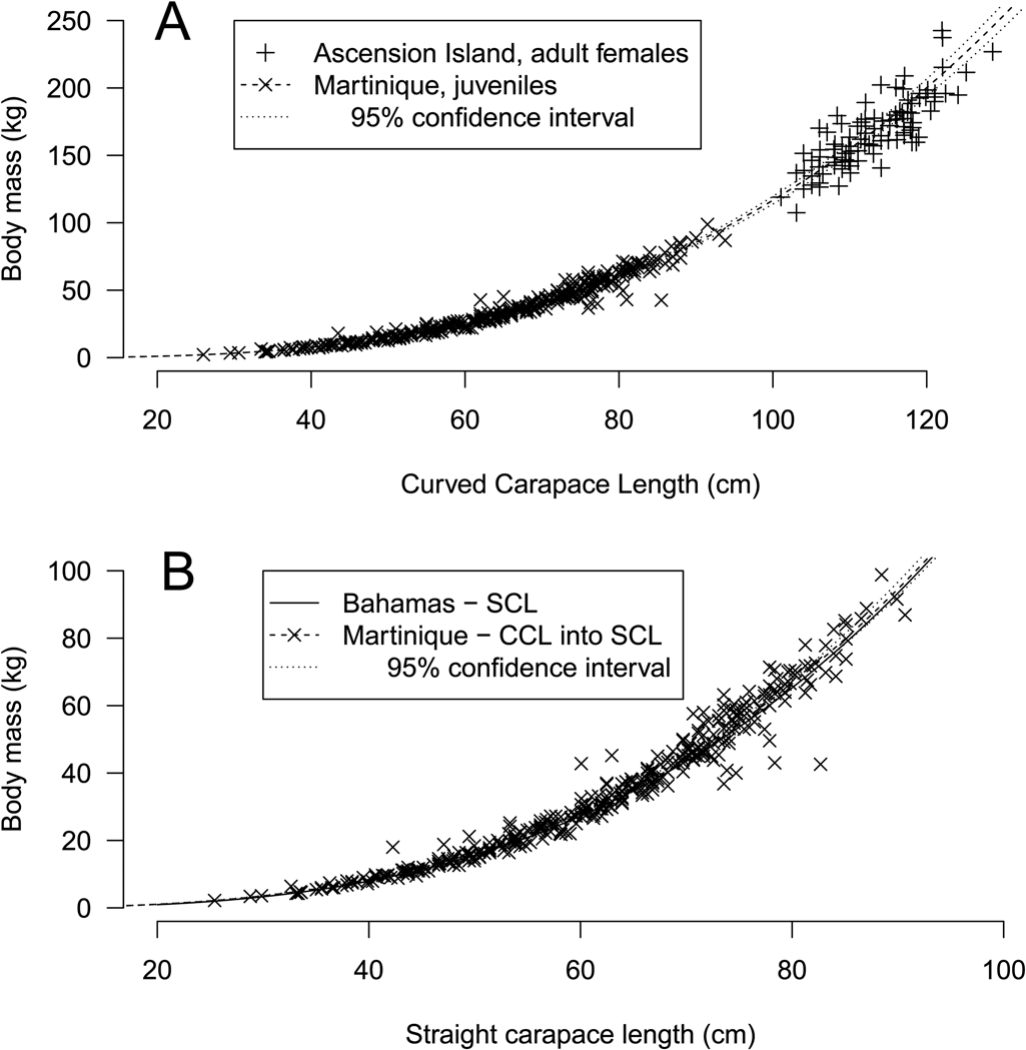
**Comparison of the relationship between BM and carapace length for (A) Ascension Island (adults) and (B) Bahamas (juveniles) against Martinique young juvenile green turtles.** In B, the two models are very similar, so the curves are superimposed. Data from Ascension and Bahamas were digitized from original publications using WebPlotDigitizer (Rohatgi, 2019).

The selected model for dataset D included only CCL and CCCW (Table 2). With this model, the average error for BM prediction was 7.09% (range 0–72.44%) (Table 3). For example, a turtle being measured weighed 50 kg and based on its measurements it would be predicted to weigh between 46.4 kg and 53.5 kg with maximum and minimum being 13.7 and 86.2 kg, respectively. The confidence interval for the relationship between BM and CCL for young juveniles in Martinique was compatible with the one observed for adults in Ascension Island (Hays et al., 2002) (Fig. 5A). Similarly, the fitted relationship between BM and SCL for juvenile green turtles in the Bahamas (Bjorndal and Bolten, 1988, 1989) was within the confidence interval for the relationship between BM and CCL for young juveniles in Martinique (Fig. 5B). However, let us recall that a significant effect of year and location was observed in our datasets. Thus, even if the relationships were similar, they could be better with these factors included in the analysis.

### Physical ecosystem characteristics

**Fig. 6.**
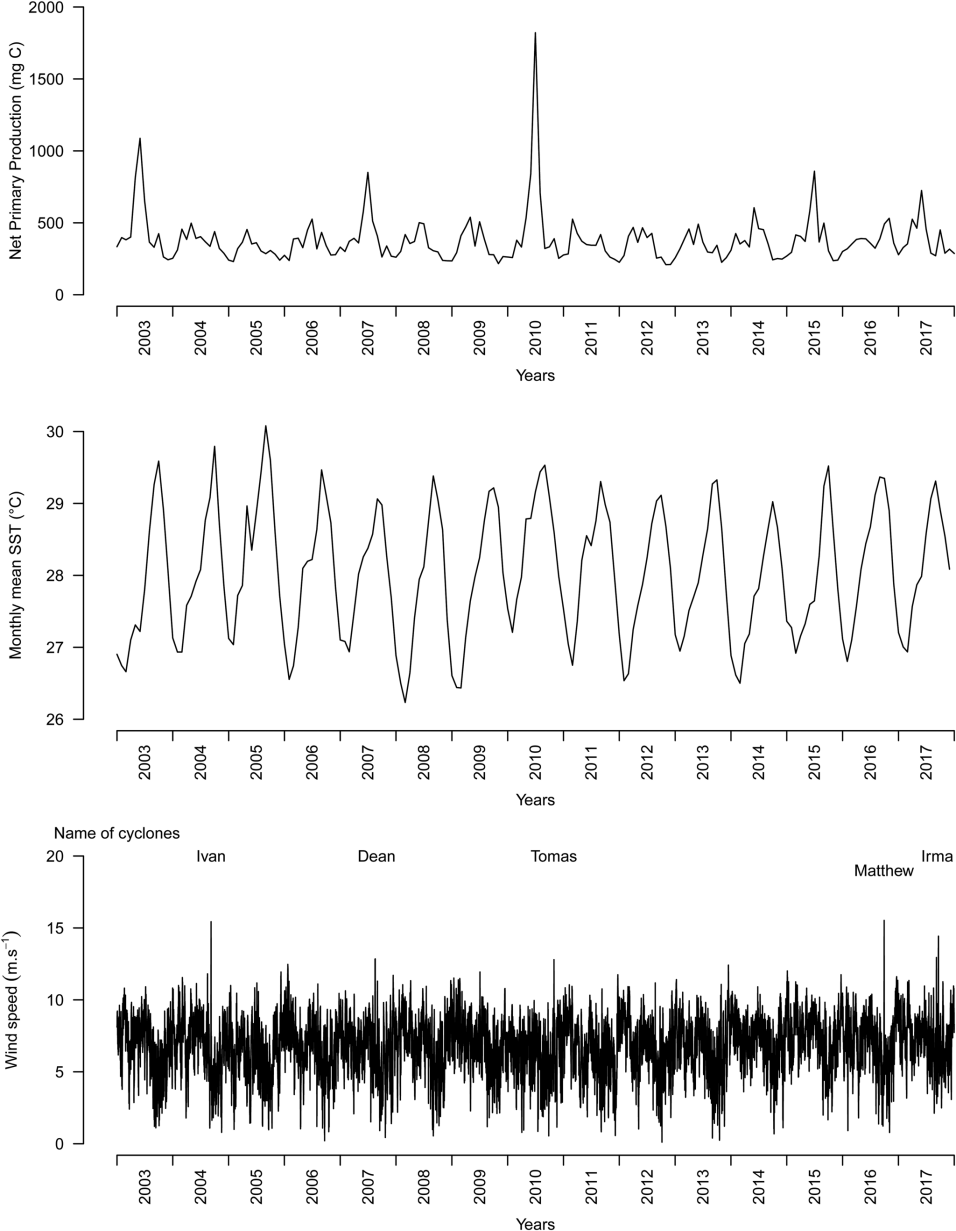
**(A) Average monthly ocean net primary production in mg C.m^−2^.day^−1^.** (B) average monthly sea surface temperature in °C, and (C) 12 h wind speed in m.s^−1^ to the west of Martinique Island (61.25 W, 14.55 N) (see location indicated by black and white circles in Fig. 2).

**Fig. 7.**
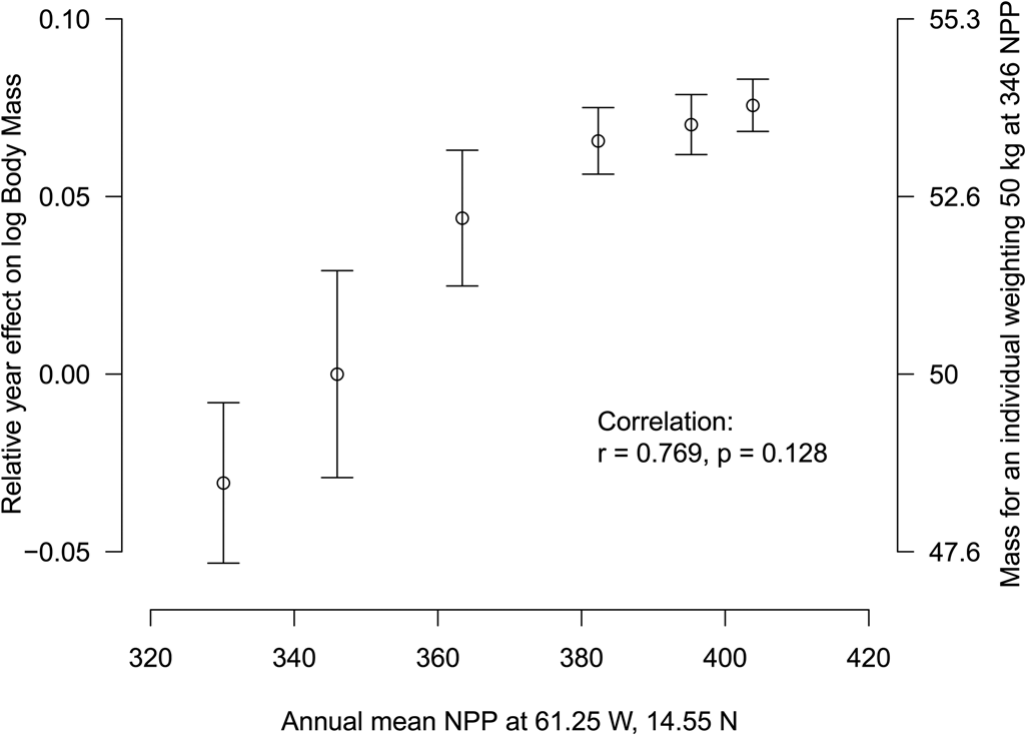
**Relationship between relative year effect on log BM versus log CCL and CCCW and year-averaged net primary production (NPP) at the west of Martinique Island (61.25 W, 14.55 N) (see location indicated by black and white circles in Fig. 2).** Bars are the quasi-standard errors (Firth and de Mezezes, 2004).

Monthly NPP and SST as well as 12 h WS are shown in Fig. 6. Correlations between year-effect for log BM versus log CCL and CCCW (see the Model for BM estimation with dataset B) was r=0.97 (*P*=0.002) for year-averaged NPP (Fig. 7), r=0.11 (*P*=0.83) for year-averaged SST and, r=0.68 (*P*=0.2) for year-maximum wind speed (*P*=0.20). A very significant positive effect of net primary production (NPP) was then noticed with heavier turtles observed for years with higher net primary production in the region.

## DISCUSSION

Our study aimed to validate the use of different body measurements to estimate BM, which can be difficult to obtain in the field, and explore the determinants of the relationship between BM and size in juvenile green turtles.

We show that using a combination of CCL, CCCW and CmidCCL and including a location and year effect, we were able to predict the BM of individuals (range 0–9.22%) with an average error of 1.70% (dataset A; Table 3). If year, location, or CmidCCL were omitted from the model, the average prediction degraded by a factor 2, while the upper range of error increased by a factor 2 (datasets B and C; Table 3). Finally, if only CCL was included in the model, the prediction of BM was considerably degraded and could reach a maximum error of 72% (dataset D; Table 3). This inexpensive (only a flexible tape measure is necessary) and non-invasive method to estimate BM based on body measurements is applicable in the field by both specialists and non-specialists. However, it is important that only trained and limited number of operators take the measurements to limit errors as already shown by Frazier (1998).

Straight line (SL) measurements are considered preferable to over-the-curve (OC) measurements for sea turtle research (Pritchard et al., 1983). In a study of juvenile green turtles, SL carapace length (SLCL) had significantly better precision (repeatability) than OCCL (Bjorndal and Bolten, 1989). Limpus (1985) recorded SL measurements to ±0.1 cm, but OC measurements to ±0.5 cm. However, SL can only be measured with large callipers, while OC measurements are much convenient in the field, especially when measurements are taken in a boat. For this reason, OC measurements, which are widely used for this species (e.g. Almeida et al., 2011; Bellini et al., 2012; Bourjea et al., 2007; Limpus, 1993), were preferred over SL in our study. Furthermore, in green turtles, Bjorndal and Bolten (1989) provide equations to convert OC into SL measurements for both carapace length and width.

In their review Wabnitz and Pauly (2008) found similar relationships between BM and body measurements in this species on a set of data including adult and juvenile individuals with a worldwide distribution. In fact, the most similar study of juvenile green turtles to be compared with the present one has been done by Bjorndal and Bolten, (1988) on a Bahamian's population. They showed a relationship between BM and carapace length of green juveniles with BM=1.07 10^−4^ CL ^3.04^, with CL being the SLCL described in Bjorndal and Bolten (1989). Using the relationship OCCL=−0.414+1.039 SLCL in Bjorndal and Bolten (1989), we were able to compare directly our data with those of Bjorndal and Bolten (1988) and show a very similar relationship between BM and carapace length (Fig. 5B). This relationship can also be extended to adult size (Fig. 5A).

We also demonstrated a year effect with individuals caught in the years 2011 and 2012 being significantly lighter than expected relative to their size. However, no difference was observed for individuals caught in the years 2015–2018 (Figs 4 and 5). We investigated for annual differences in physical oceanographic conditions (Fig. 6) close to the capture sites (Fig. 2). A very significant relationship for this pattern in terms of the net primary production (Fig. 7) was detected but not for sea surface temperature or occurrence of cyclones: turtles are heavier relative to their linear dimensions for years with high net primary production. Whereas it seems logical for a herbivorous animal that the higher the net primary production the heavier the animals, this effect was never demonstrated before. This most likely exists only in juveniles as for adults there will be massive changes in BM depending on where an individual is in its breeding cycle (e.g. just about to breed or just completed breeding). So for an adult the BM probably varies by several 10s of kg over the breeding cycle (Hays and Scott, 2013). Also, we demonstrated a south-north effect with individuals caught in the north being lighter than expected relative to their size (Fig. 1A). This very local pattern (<10 km) is surprising as no spatial effect or spatio-temporal interaction was observed in West Atlantic hawksbill growth rates inhabiting the same region (Bjorndal et al., 2016). It should be noted that individuals are very faithful to their habitat, being captured in the same bay from year to year. This pattern does not appear to be linked to the density of individuals recorded in the different bays (Fig. 1B). Thus, it would be expected that marine productivity could vary between the bays with a north-south or annual pattern, but this remains to be investigated. Other hypotheses may also explain these differences; for example, human pressure influencing the quality of bays in terms of resources, differences in currents, and the global impact of cyclones in the south versus the north.

Nevertheless, these results highlight that the environmental and nutritional quality of specific local habitats should be further explored in all bays frequented by green turtles in order to develop rational management and conservation plans at the territorial scale of Martinique Island.

## MATERIALS AND METHODS

### Ethics statement

This study meets the legal requirements of the countries in which this work was carried out and follows all institutional guidelines. The protocol was approved by the ‘Conseil National de la Protection de la Nature’ (CNPN, http://www.conservation-nature.fr/acteurs2.php?id=11), and the French Ministry for Ecology, Sustainable Development, and Energy (permit number: 2013154-0037), which acts as an ethics committee in Martinique. After the evaluation of the project by the CNPN, fieldwork was conducted in strict accordance with the recommendations of the Police Prefecture of Martinique in order to minimise the disturbance to the animals (authorisation: n**°**201505-0002).

### Animal capture

Turtles were captured in October 2011–2012 and 2015–2018 at Grande Anse d'Arlet, Anse du Bourg, Anse Dufour, Anse Noire, Le Carbet and Le Prêcheur, all located in Martinique Island in the eastern Caribbean Sea (Fig. 2). The surface of these bays was estimated from satellite pictures using the surface from the shoreline to the straight line linking the two edges of the bays. Catches were performed between 8 h and 17:00 h at a depth ranging from 2 m to 15 m. When turtles were static, i.e. resting or feeding (head down) on the sea floor, they were caught by a free diver who discreetly dived close to the head of the turtle to avoid detection. Once close enough and above the animal, the free diver seized the nuchal and pygal areas of the shell. They then positioned the turtle against their chest, keeping its anterior flippers against his breastplate, and rose to the surface. A second free diver held the fore flippers and helped lift the turtle into a boat for body measurements and tagging.

### Data collection

We recorded the date, hour, tag number and place of capture for each turtle. The presence of a passive integrated transponder (PIT) was also recorded; in its absence, a PIT (ID-100, TROVAN^®^) was injected into the right tricep. The number was checked using a manual reader (GR250, TROVAN^®^). The health status of each animal was also recorded, particularly in the presence of a visible external tumour. Each animal was measured (see below) with a flexible measuring tape (±0.1 cm). Only two trained operators (M.B. and D.C.) measured the animals. Measurement differences were less than 1% for these two operators.

**Fig. 8.**
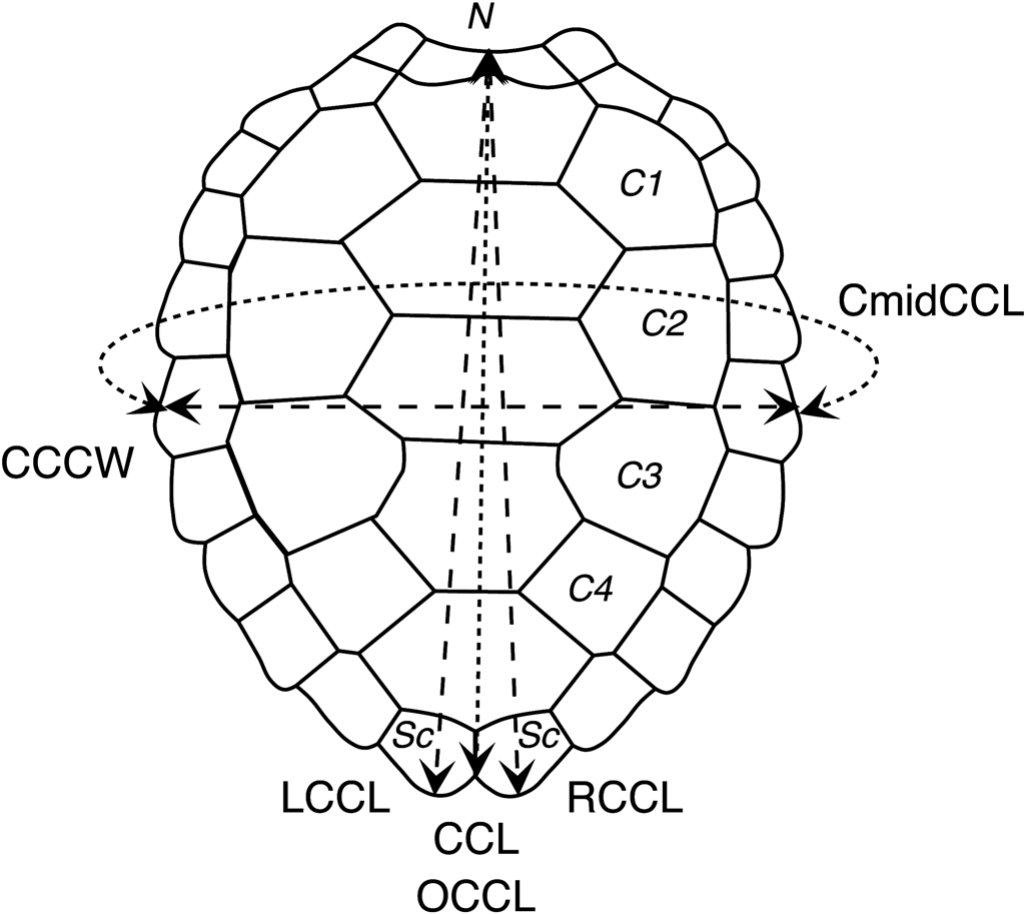
**Schematic illustration of measurements.** Scutes: Sc, supracaudal; N, nuchal; C, costal. Measurements: CCL, curved carapace length; equivalent of OCCL in Bjorndal and Bolten (1989); RCCL, right curved carapace length; LCCL, left curved carapace length; CCCW, central curved carapace width; CmidCCL, circumference at mid curved carapace length.

We measured CCL (measured between nuchal and supracaudal scutes) and CCCW (measured between left and right costal scutes 2 and 3) (Bolten, 1999). Bjorndal and Bolten (1989) OC carapace length (OCCL) is equivalent to our CCL measurement (Fig. 8). In addition to these standard measurements, we also recorded the left (LCCL) and right curved carapace length (RCCL) from the mid-point of the nuchal scute to the left or right supracaudal scute. A comparison of CCL, RCCL and LCCL allows measurement errors to be detected. However, because the marginal points of supracaudal scutes are susceptible to breakage and differential wear, CCL remains the most accurate measurement (Bjorndal and Bolten, 1989). For this reason, in our predictive models, we chose to use only CCL and so discarded LCCL and RCCL measurements. Circumference at mid-CCL (CmidCCL) was also recorded in approximately one-third of captures (Fig. 8). Finally, the BM of individuals was recorded using an electronic crane scale (maximum mass 600 kg±0.1 kg, Kern, HUS600K Model) before their release at sea in the same location. About 10% of individuals were measured and weighed more than once during field work. The electronic crane scale was suspended from a beam. A hammock, used to hold the turtles during weighing, was attached at the electronic crane scale which was then tared to ‘0’ before each turtle's weighing.

Bjorndal and Bolten (1988) estimated juvenile green turtle BM in the Bahamas based on SL measurements. Bjorndal and Bolten (1989) also gave equations to convert OC into SL measurements for both the carapace length and width of juvenile green turtles. We used these equations to compare their estimates of BM with our own.

### Statistical analyses

Statistical analyses were carried out using R software version 3.6.1 (R Core Team 2019). For this study, no wounded (fin cut), or sick individuals (presence of fibropapilloma tumours) were integrated in the analysis.

To test the effect of some morphological and time-location parameters on the accuracy of the mass prediction, four different datasets were built: (A) a dataset with BM, CCCW, CCL, CmidCCL, year, location and the identity of the animal; (B) a dataset with BM, CCCW, CCL, year, location and the identity of the animal; (C) a dataset with BM, CCCW, CCL and the identity of the animal; (D) a dataset with BM and CCL.

Dataset B allowed us to test the precision of the BM estimation without CmidCCL as it can be difficult to measure and was only recorded in one-third of captures. Dataset C was a minimal dataset in case the location and year were not be available. Finally, dataset D was used to compare our data with other published analyses for this species (Bjorndal and Bolten, 1988, 1989; Hays et al., 2002). For this former analysis, when an individual was measured and weighed on several occasions, only the first measurement was used. Data were then analysed using a linear model without random individual effect so as to have similar conditions as previous studies. Only the individuals with a complete set of information within a dataset were retained for analyses. All measurements were log-transformed to limit the effect of heteroskedasticity. Year was always treated as a categorical variable.

About 10% of individuals were measured and weighed more than once during field work. A mixed model with individual as the random effect and Gaussian distribution for measurements was then chosen. Restricted maximum likelihood was used as a fit criterion to ensure unbiased variance. Model selection was performed using the conditional Akaike information criterion (cAIC). This measure of the quality of fit penalised by the number of parameters corrected (Burnham and Anderson, 2002) was specially developed for mixed models (Greven and Kneib, 2010; Säfken et al., 2018 preprint). A backward model selection using cAIC was used and then stopped when the most complex model was selected. Factors were removed one at a time. A parameter involved in an interaction was never removed from the analysis. Model selection was stopped when the most complex model was selected based on cAIC.

Quasi-variances (and corresponding quasi-standard errors) for estimated model coefficients relating to the levels of a categorical explanatory variable (years and locations) were estimated using the method of Firth and de Mezezes (2004) that is specifically adapted for generalised linear mixed models.

The equation of the percentage of errors for one individual is thus:
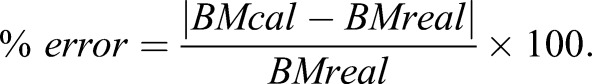
With *BMcal* being BM estimated using the selected model and *BMreal* being the BM determined by direct weighing.

### Physical ecosystem characteristics

To link year effect with physical oceanography, ocean net primary production (NPP), sea surface temperatures (SST) and wind speed (WS) were obtained from public databases for the location closest to the capture bays. NPP is commonly modelled as a function of chlorophyll concentration and is based on the original description of the vertically generalised production model (VGPM) (Behrenfeld and Falkowski, 1997), MODIS surface chlorophyll concentrations (Chlsat), MODIS 4-micron sea surface temperature data (SST4), and MODIS cloud-corrected incident daily photosynthetically active radiation (PAR). Euphotic depths were calculated from Chlsat following Morel and Berthon (1989). NPP was compiled from the Ocean Productivity website (http://www.science.oregonstate.edu). SST and WS were obtained from the European Centre for Medium-Range Weather Forecasts database (https://www.ecmwf.int). WS was calculated from the two orthogonal WS vectors *u* and *v* using 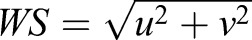.
